# Speech and language classification in the human phenotype ontology

**DOI:** 10.1038/s41431-024-01635-6

**Published:** 2024-07-01

**Authors:** Angela T. Morgan, Ben Coleman, Adam P. Vogel, Alisdair McNeill, Peter N. Robinson

**Affiliations:** 1 Murdoch Children’s Research Institute, Melbourne, Australia; 2 Speech Pathology, School of Health Sciences, University of Melbourne, Melbourne, Australia; 3 The Jackson Laboratory for Genomic Medicine, Farmington, Connecticut USA; 4 Division of Neuroscience & Neuroscience Institute, University of Sheffield, Sheffield, United Kingdom; 5 Berlin Institute of Health at Charité - Universitätsmedizin Berlin, Berlin, Germany

**Keywords:** Gene ontology, Human behaviour

## Introduction

Communication is a critical skill, not only for daily survival but also for leading a successful and fulfilled life. Speech and language skills are supported by widespread, complex brain networks. Ergo, communication disorders are common in neurogenetic conditions and may appear even with relatively subtle perturbations of brain development [1–4]. Speech and language difficulties frequently co-occur but are distinct and dissociable skills. In simple terms, speech is the perception and motor production of sounds; and language the ability to understand and produce a message using vocabulary and grammar, in spoken or written form.

The human genetics literature is currently restricted by conflation of the terms speech and language, and by a lack of specificity of the sub-phenotypes of these domains. This is reflected in the Human Phenotype Ontology (HPO) which has grown organically with speech and language terms inputted by experts working across multiple conditions, rather than being developed with a pre-determined framework. The HPO provides a standardized vocabulary of phenotypic abnormalities of human disease, with each term describing a specific feature, such as ‘language impairment’. The HPO contains over 16,000 terms and over 156,000 annotations to hereditary diseases. The HPO is a flagship of the NIH-supported Monarch Initiative; dedicated to semantic integration of biomedical and model organism data with a view to improve research. Software has been developed by the HPO project and others to support this goal, facilitating phenotype-driven differential diagnostics, genomic diagnostics, and translational research.

Speech and language classification in the HPO has detailed descriptors in some areas, and in others, a blunt selection of phenotypes which fail to dissociate speech and language features across genotypes, where presentations are in fact, different. A common example of this occurs in neurodevelopment where a catch-all-term of *Delayed speech and language development HP:0000750* is commonly applied across conditions, even those that are strikingly different in the clinic, e.g. individuals with *FOXP2*-only disorder are typically verbal communicators with a relatively homogeneous presentation of childhood apraxia of speech [5], in comparison to individuals with *KAT6A* syndrome where around 75% of individuals remain minimally verbal and rely on aided communication even into adulthood [6]. Documenting the natural history of specific speech and language phenotypes for distinct conditions paves the way for application of targeted therapies at an early age [7–9]; critical for optimising communication, wellbeing, and life outcomes. A final challenge of the current limited speech and language classification in the HPO is an inability to capitalise on rich electronic medical record data to drive knowledge discovery across diagnosis, prognosis or therapies in the speech and language genetics field. Here we specify core challenges to be addressed and suggest an approach to improve the specificity and efficiency of speech and language ontologies with the HPO.

## Current speech and language phenotyping in the HPO

**Fig. 1:**
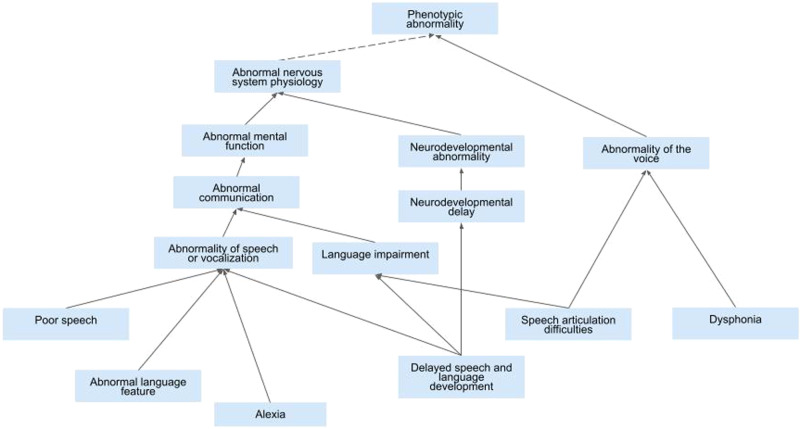
Current speech and language terminology branching in the Human Phenotype Ontology (HPO). Blue boxes denote examples of the broad and often overlapping phenotyping options related to speech and language phenotyping in the HPO. Not all options are presented here.

The HPO successfully incorporates a broad range of speech and language phenotypes across the ontology. Yet inconsistencies of the system contribute to clinical confusion and poor application of terms. There are currently three core pathways by which to reach speech and language phenotypes (see Fig. 1). Two pathways stem from *Abnormality of the nervous system HP:0000707, Abnormal nervous system physiology HP:0012638*. One pathway branches to *Abnormality of mental function HP:0011446* and *Abnormal communication HP:0034434* where six further communication sub-phenotypes are denoted. The second pathway from *Abnormal nervous system physiology HP:0012638* branches to *Neurodevelopmental abnormality HP:0012759*, *Neurodevelopmental delay HP:0012758* and *Delayed speech and language development HP:0000750* which results in three further language and non-verbal sub-phenotypes. The third and final pathway branches from *Abnormality of the voice HP:0001608* under which there are nine further categories largely focused on speech, voice and resonance phenotypes. There are also further pathways focused on social language which we will not discuss in detail here. These multiple pathways, all resulting in different end descriptors, lead to confusion for busy clinicians who tend to find their own most efficient, yet arguably not always most specific, pathway for their conditions of interest.

A further challenge regards inconsistent application of medical terminology. One example is the use of a- versus dys-, the former denoting a complete absence of a skill and the latter a relatively less impaired presentation. There are inconsistencies in application of terms a-phasia, dys-phasia, a-narthria, dys-arthria, a-graphia, dys-graphia across the medical literature and these terms are reflected in the HPO. For example, the term a-phasia is now unanimously used in the adult language literature, not dys-phasia. Further, aphasia was originally defined as an acquired language impairment following stroke, but it is now also used to categorise language in neurodegenerative diseases such as primary progressive aphasia, not just acquired conditions. Hence there is a need for all these synonymous or confusing terminological issues to be refined in a clear framework with transparency of approach and definitions to best support their clinical application.

## Refining classification of speech and language in the HPO

**Fig. 2:**
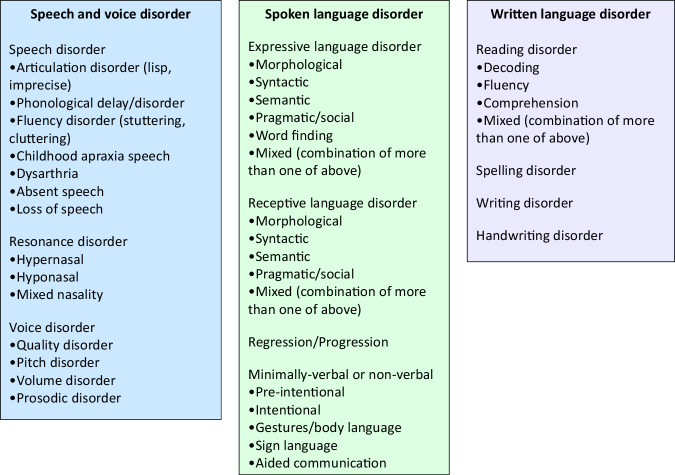
Example approach for an overarching speech and language classification in the Human Phenotype Ontology. Adapted from American Speech Hearing Association, World Health Organisation, Royal College of Speech Language Therapists, International Association for Communication Disorders and Sciences, Speech Pathology Australia, DSM-5, ICD-11, National Institute of Child Health and Human Development.

Speech and language and associated disorders can be classified in myriad ways. Further to the HPO, core medical classifications with speech and language terms include the World Health Organisation’s International Classification of Disease’s (ICD-11) and the American Psychiatric Association’s Diagnostic and Statistical Manual of Mental Disorders (DSM-V). These classification systems are, necessarily, exceptionally broad and as a result they have a relatively limited specificity for speech and language diagnoses (e.g., just five diagnostic categories under communication disorder for children across both domains of speech and language in the DSM-V) and often focus on literacy as a separate domain without the ability to consider links between speech, language, and literacy. The other challenge for adoption of some existing large-scale systems is the common division of paediatric and adult populations, as currently also occurs in the HPO, with age-related terminology commonly applied in each case. For example, use of the term ‘language’ in children in contrast to the term ‘aphasia’ (traditionally applied to acquired language disorders) used for adults. Failure to take a ‘lifespan approach’ to terminology wherever possible, particularly in the context of neurogenetic developmental disorders, results in complexities when collating data to gain further insights into the genetic condition of interest, e.g. if one were to attempt to map the language abilities of individuals with an *EBF3-*neurodevelopmental disorder longitudinally from birth into adulthood using HPO terms mined from electronic medical records. Arguably a parsimonious approach has been adopted by leading clinically related speech and language organisations or peak bodies, such as the American Speech and Hearing Association, Speech Pathology Australia, the Royal College of Speech and Language Therapists and the World Health Organisation. A classification adapted from these established clinically relevant frameworks includes core domains of speech as well as spoken and written language and their sub-phenotypes (Fig. 2, Supplementary Tables).

Speech involves perception of speech sounds as well as the use of the articulators, namely lips, larynx, soft and hard palate, tongue, jaw and cheeks, to produce a speech signal, made up of the sounds of one’s language. Speech also requires adequate airflow from the lungs to pass through the larynx with enough force to produce voicing and to control the loudness and prosodic aspects of speech. The size, placement and configuration of an individual’s larynx also impacts pitch for speech, whether too high, low or sex or age appropriate. The velum or soft palate must move quickly and rapidly to produce oral or nasal (m, n, ng in the case of English) sounds. Speech challenges can result due to speech perception difficulties (e.g., hearing loss), structural deficits (e.g., cleft palate, macroglossia, malocclusion) or neurological deficits (e.g., hypotonia of face, tongue etc, soft palate poor function, vocal fold palsy, cerebellar disease). Numerous pathologies of speech are already encapsulated within the HPO, yet these could arguably be better organised under an easy to apply framework which guides the clinician and encourages specificity in use of the terms speech and language as a constructive first step.

Language, by contrast to speech, is a higher-level cognitive construct and can be divided further into spoken and written modalities, with the latter also encompassing literacy. Spoken and written language both involve imparting and receiving a message – that is they involve both expressive and receptive channels of communication. Beyond expressive and receptive sub-domains, language can be further categorised into sub-phenotypes affecting syntactic or semantic domains, minimally verbal presentations or even regression/disease progression. Language features across children and adults are highly similar, yet different terminology has been historically used, which could also be better addressed in a new system to better fit the many lifelong neurogenetic conditions that exist in our society. It is important to note that we are not advocating for an immediate change to use of such a classification system as shared here in Fig. 2. Rather we are using this viewpoint article to highlight current challenges and to advocate for positive change. The current authorship team are leading a working group to carefully consider and revise the HPO speech and language classification and branching of terms using a consensus-based approach with other international experts in the field.

## Summary

Here we propose a revision and simplification of the speech and language hierarchy and sub-phenotypes. Application of an over-arching framework with clarification of the differences between speech and language domains and their sub-phenotypes will transform current communication phenotyping. In turn, increased phenotypic precision will improve individual clinical care, enable a more sensitive understanding of similarities and differences across genetic conditions to drive efficiencies of treatment and provide a solid platform for data driven discovery in the speech and language genetics field.

## Supplementary information

Speech and language phenotyping in the Human Phenotype Ontology

## References

[1] Liégeois FJ, Turner SJ, Mayes A, Bonthrone AF, Boys A, Smith L, et al. Dorsal language stream anomalies in an inherited speech disorder. Brain. 2019;142:966–77. 30796815 10.1093/brain/awz018

[2] Liégeois FJ, Hildebrand MS, Bonthrone A, Turner SJ, Scheffer IE, Bahlo M, et al. Early neuroimaging markers of FOXP2 intragenic deletion. Sci Rep. 2016;6:35192. 27734906 10.1038/srep35192 PMC5062117

[3] Thompson-Lake DGY, Scerri TS, Block S, Turner SJ, Reilly S, Kefalianos E, et al. Atypical development of Broca’s area in a large family with inherited stuttering. Brain. 2022;145:1177–88. 35296891 10.1093/brain/awab364 PMC9724773

[4] Morgan AT, Scerri TS, Vogel AP, Reid CA, Quach M, Jackson VE, et al. Stuttering associated with a pathogenic variant in the chaperone protein cyclophilin 40. Brain. 2023;146:5086–97. 37977818 10.1093/brain/awad314 PMC10689913

[5] Morison LD, Meffert E, Stampfer M, Steiner-Wilke I, Vollmer B, Schulze K, et al. In-depth characterisation of a cohort of individuals with missense and loss-of-function variants disrupting *FOXP2*. J Med Genet. 2023;60:597–607. 36328423 10.1136/jmg-2022-108734 PMC10314088

[6] St John M, Amor DJ, Morgan AT. Speech and language development and genotype-phenotype correlation in 49 individuals with KAT6A syndrome. Am J Med Genet A. 2022;188:3389–3400. 35892268 10.1002/ajmg.a.62899

[7] Braden RO, Amor DJ, Fisher SE, Mei C, Myers CT, Mefford H, et al. Severe speech impairment is a distinguishing feature of FOXP1-related disorder. Dev Med Child Neurol. 2021;63:1417–26. 34109629 10.1111/dmcn.14955

[8] Morison LD, van Reyk O, Forbes E, Rouxel F, Faivre L, Bruinsma F, et al. CDK13-related disorder: a deep characterization of speech and language abilities and addition of 33 novel cases. Eur J Hum Genet. 2023;31:793–804. 36599938 10.1038/s41431-022-01275-8 PMC10325997

[9] Morgan A, Braden R, Wong MMK, Colin E, Amor D, Liégeois F, et al. Speech and language deficits are central to SETBP1 haploinsufficiency disorder. Eur J Hum Genet. 2021;29:1216–25. 33907317 10.1038/s41431-021-00894-x PMC8384874

