# Supplementary material for: Speech and language classification in the human phenotype ontology
Source: Eur J Hum Genet. 2024 Jul 1;32(12):1518–21. doi: 10.1038/s41431-024-01635-6 (PMC11607328; doi:10.1038/s41431-024-01635-6)
Supplement: Supplementary file 1 — Speech and language phenotyping in the Human Phenotype Ontology [file 41431_2024_1635_MOESM1_ESM.docx]

**Supplemental tables: Speech and language phenotyping in the Human Phenotype Ontology**

| **Table 1A. Speech and voice disorder** | | |  |
| --- | --- | --- | --- |
| **Speech disorder** |  | Deficit in the verbal production of meaningful speech sounds of language relative to peers. May manifest as a range of sub-phenotypes, e.g. articulation disorder, phonological delay/disorder, CAS, stuttering, etc. | |
|  | Articulation disorder | Consistent distortion of one or more speech sounds, commonly a lisp or de-rhotacization (/r/ sound is unclear) due to a mislearned/inaccurate movement plan, or due to structural causes (e.g. missing front teeth, cleft palate, macroglossia). | |
|  | Apraxia | Disorder of motor programming/planning. Inconsistent production of consonants and vowels across repeated productions. Lengthened and impaired coarticulatory transitions between sounds and syllables (e.g., omissions of sounds, vowel errors, repetitions).Inappropriate prosody/disrupted intonation, e.g. placing stress on a typically unstressed syllable or using equal stress across all syllables. | |
|  | Dysarthria | Disorder of neuromuscular control and tone, e.g., spasticity, ataxia, fluctuating tone, weakness, involuntary movements. This results in imprecision of speech due to impairments in one or more areas of phonation, articulation, prosody, resonance, respiration. Variable manifestation depending on underlying impairment, e.g., slurred speech, slow rate, poor voice quality such as hoarseness or breathiness, hyper or hypo-nasal speech, prolonged pauses in speech, challenges using the correct pitch tone and volume. Distinct from apraxia as individual speech sound errors are typically more consistent in nature but may be markedly imprecise or distorted. Affects intelligibility and/or naturalness. | |
|  | Stuttering | Disorder of speech fluency characterized by repetitions (of sounds, syllables, words and/or phrases), prolongation of sounds, and hesitations and/or blocks. | |
|  | Cluttering | Disorder of speech fluency characterized by some repetitions (of sounds, syllables, words and/or phrases), but speech also rushed. Patients describe ‘tripping’ over words and an inability to slow their speech. | |
|  | Absent speech | Speaker does not use any verbal speech. See Minimally verbal. | |
|  | Loss of speech | See Regression/progression, under Minimally verbal, Spoken language. | |
|  | Resonance disorder | Disorder of oral and nasal contrasts for speech. Speech will have altered nasal resonance, being perceived as either hypernasal, hyponasal or mixed in nature. May be part of dysarthria and only referred to separately if no other dysarthric features. | |
| **Voice disorder** |  | Deficit of the voice in terms of quality, pitch, volume, relative to one’s developmental age, gender and culture. As an isolated voice disorder this is distinct from dysarthria which typically affects more than one subsystem. | |
|  | Quality | Inappropriate quality of the voice, whether raspy, hoarse, weak, tremulous, strained, etc. May be due to vocal cord paralysis, lesions (nodules, polyps, cysts), spasms or poor control of the larynx. | |
|  | Pitch | Inappropriate pitch of the voice, whether excessively high or too low. | |
|  | Volume | Inappropriate loudness of the voice, whether excessively loud or too quiet. | |
|  | Prosody | Inappropriate prosody of the voice in relation to the rhythm of speech. | |

| **Table 1B. Spoken Language Disorder Phenotypes** | | |
| --- | --- | --- |
| Spoken language disorder |  | Deficit in production (expressive language) or comprehension (receptive language) of language relative to peers. One language area or a combination of areas may be implicated (e.g. expressive, receptive, semantics, syntax, word finding, as below). |
| Expressive language disorder |  | Deficit in production or output of language relative to peers. One language area or a combination of areas may be implicated (e.g. semantics, syntax, word finding, as below). |
|  | Phonological delay or disorder | Predictable rule-based speech errors. Delay or disorder in understanding and/or use of speech sounds to convey meaning. e.g. “backing” pattern says *k*ey for *t*ea, or *g*og for *d*og; using posteriorly produced sounds (k, g) in place of anteriorly produced sounds (t, k). |
|  | Morphological disorder | Incorrect use of morphological terms of a language to denote past tense, future tense, plurals (e.g. walked vs walking; man vs men). |
|  | Syntactic disorder | Spoken difficulty of sequencing words, thoughts, and information in right order using the correct parts of language. May omit words in sentences. Limited number of complex sentences for age / cognitive status. |
|  | Semantic disorder | Difficulties learning, using and recalling new and known words, categorizing words (e.g. an apple - fruit), describing and comparing words or concepts, using adjectives (shape, colour, size, feelings). |
|  | Word finding | Impaired spontaneous recall of known words with no impairment of comprehension or capacity to repeat the words. Also known as anomia. May be a semantic issue or a visual processing issue if picture naming. May be able to produce the word with use of phonemic or semantic cues. |
|  | Pragmatic/social language disorder | Disorder of using spoken language rules appropriately according to the social context. Involves appropriate use of social rules (e.g. turn taking), facial expression, gestures, use and maintaining appropriate topics of conversation, non-verbal eye contact or gestures. |
|  | Discourse | An impaired ability to produce coherent narratives, maintaining a topic, or using appropriate grammar and syntax in extended speech events such as conversations or monologues. Deficits relate to the flow of conversation, the use of appropriate vocabulary, the coherence and cohesion of concepts, and the pragmatic aspects of language use (like turn-taking and appropriateness in different social contexts). Difficulties in this domain impact a speaker’s ability to manage real-life communicative tasks, such as conversing, storytelling, or explaining. |
| Receptive language disorder |  | A deficit in the comprehension of language relative to peers. A single domain or combination of domains of language may be implicated (e.g. syntax, morphology, as below). |
|  | Morphological disorder | Disorder of understanding the morphological terms of a language to denote past tense, future tense, plurals (e.g. walked vs walking; man vs men). |
|  | Syntactic disorder | Disorder of understanding the meaning and order of words, thoughts and information in spoken sentences. May understand only simplified sentences or commands for age. |
|  | Semantic disorder | Disorder of understanding word meaning and the relations between words such as being able to categorize words (e.g. sheep is an animal). |
|  | Pragmatic/social language disorder | Disorder of understanding language rules according to the social context. Involves reading appropriate social cues such as facial expression (e.g. when someone is bored or angry), interpreting tone of voice (e.g. sarcasm, distress), known when to take turns in conversation, using an appropriate level of familiarity, misinterpreting higher-level language phrases that require interpretation (e.g. ‘it’s raining cats and dogs’; ‘I’m over the moon’). |
| Regression / Progression |  | A loss of language skills already mastered by the speaker, e.g. stops using previously learned words of speech sounds; loses social interaction skills; loses ability to engage others with gesture, pointing. Children may revert from simple words to babbling. Adults may use less sophisticated vocabulary, lack cohesion in arguments or become less verbal. |

| Table 1C. Minimally verbal speech and language phenotypes | | |
| --- | --- | --- |
| **Minimally verbal** |  | Relies on non-verbal communication such as gestures, uses < 50 single spoken words (not combining words), pre-symbolic or vocalisations only. |
|  | Pre-intentional communication | Involuntary (pre-symbolic) communication behaviours used to express emotion (e.g. comfort, discomfort, hunger, tired). Behaviours interpreted by caregivers to identify child’s wants and needs. Example behaviours include:  reaching or eye gaze towards desired person/object; head turning away to show disinterest; facial expressions like smiling for enjoyment; body movements such as bouncing for joy or happy anticipation; yawning when sleepy; crying to express discomfort; cooing/laughing to show joy; loud vocalization/scream to show dislike. |
|  | Intentional prelinguistic communication | Intentional (symbolic) prelinguistic communication behaviours voluntarily used before the development or reliance on verbal speech to express emotion, wants and needs. Example behaviours include: nodding head to request an item, shaking head to refuse an item, smiling, waving, pointing to give or receive, playful noises such as brmmm for a car or moo for cow, laughing to show joy. |
|  | Gestures/body language | Gestures and body language are less formal and typically non systematized non-verbal communication forms. |
|  | Sign language | A formal, agreed-upon set of movements used to form a non-verbal language. |
|  | Aided communication | Aided communication uses equipment, either low-tech (no power) or hi-tech (need power or batteries) or both, to help the individual communicate. Often use pictures and symbols as well as words. Low-tech examples include: alphabet and word boards; pen and paper; communication books with pictures, photos or symbols. High-tech examples include eye gaze technology and voice generating tools. |
| **Regression / Progression** |  | A loss of language skills already mastered by the speaker, e.g. stops using previously learned words of speech sounds; loses social interaction skills; loses ability to engage others with gesture, pointing. Children may revert from simple words to babbling. Adults may use less sophisticated vocabulary, lack cohesion in arguments or become less verbal. |

| **Table 1D. Written language disorder phenotypes** | | |
| --- | --- | --- |
| **Written language disorders** |  | Deficit in reading (decoding, fluency, comprehension), spelling or written expression relative to peers. |
| **Reading disorders** |  |  |
|  | Decoding | Deficit in identifying words when reading, either through word decoding (using sounds) or sight word identification. |
|  | Fluency | Deficit in recognizing and reading words within a text as regards accuracy, speech, rhythm, and intonation. |
|  | Comprehension | Deficit in ability to understand the meaning of written text. Includes semantic, syntactic knowledge, executive function skills. |
| **Spelling** |  | Deficit in mapping from a sound to a letter to spell a word in written form. May encompass difficulties with phonological, orthographic knowledge, mental grapheme representations, mental orthographic images, semantic knowledge, morphological knowledge. |
| **Writing** |  | Deficit in ability to use letters of language to produce words and convey ideas. Includes planning, drafting, revising, and editing text; writing appropriately to the audience and conveying the purpose of the text. |
| **Handwriting** |  | Deficits in ability to write, express thoughts in writing, and complete writing efficiently. Linked to deficits in orthographic coding, i.e. mapping abstract representation of letters to motor movements to write words or motor impairments, e.g. ataxia. |
